# Luteolin inhibits progestin-dependent angiogenesis, stem cell-like characteristics, and growth of human breast cancer xenografts

**DOI:** 10.1186/s40064-015-1242-x

**Published:** 2015-08-22

**Authors:** Matthew T. Cook, Yayun Liang, Cynthia Besch-Williford, Sandy Goyette, Benford Mafuvadze, Salman M. Hyder

**Affiliations:** Department of Biomedical Sciences, University of Missouri, Columbia, MO 65211 USA; Dalton Cardiovascular Research Center, University of Missouri, 134 Research Park Drive, Columbia, MO 65211 USA; IDEXX BioResearch, Columbia, MO 65202 USA

**Keywords:** Luteolin, Breast cancer, Tumor growth, Medroxyprogesterone acetate, Therapeutic

## Abstract

**Purpose:**

Clinical trials and epidemiological evidence have shown that combined estrogen/progestin hormone replacement therapy, but not estrogen therapy alone, increases breast cancer risk in post-menopausal women. Previously we have shown that natural and synthetic progestins, including the widely used synthetic progestin medroxyprogesterone acetate (MPA), increase production of a potent angiogenic factor, vascular endothelial growth factor (VEGF), in human breast cancer cells, potentially providing an explanation for progestin’s mechanism of action. Here, we tested the effects of luteolin (LU), a flavonoid commonly found in fruits and vegetables, on inhibiting progestin-dependent VEGF induction and angiogenesis in human breast cancer cells, inhibiting stem cell-like characteristics, as well as breast cancer cell xenograft tumor growth in vivo and expression of angiogenesis markers.

**Methods:**

Viability of both T47-D and BT-474 cells was measured using sulforhodamine B assays. Enzyme-linked immunosorbent assays were used to monitor VEGF secretion from breast cancer cells. Progestin-dependent xenograft tumor growth was used to determine LU effects in vivo. CD31 immunohistochemistry was used to determine blood-vessel density in xenograft tumors. CD44 expression, aldehyde dehydrogenase activity, and mammosphere-formation assays were used to monitor stem cell-like characteristics of breast cancer cells.

**Results:**

Luteolin treatment reduced breast cancer cell viability, progestin-dependent VEGF secretion from breast cancer cells, and growth of MPA-dependent human breast cancer cell xenograft tumors in nude mice. LU treatment also decreased xenograft tumor VEGF expression and blood-vessel density. Furthermore, LU blocked MPA-induced acquisition of stem cell-like properties by breast cancer cells.

**Conclusions:**

Luteolin effectively blocks progestin-dependent human breast cancer tumor growth and the stem cell-like phenotype in human breast cancer cells.

## Background

Breast cancer is the most commonly diagnosed form of cancer and the second-leading cause of cancer-related death in American women. In 2015, an estimated 232,000 new cases of breast cancer will be diagnosed, with approximately 40,000 deaths (Siegel et al. 2015). A subset of both newly diagnosed cases and breast cancer-related deaths is linked to the use of hormone replacement therapy (HRT) containing a combination of estrogen and progestin in post-menopausal women (Ross et al. 2000; Chlebowski et al. 2003). A progestin component is administered to women with an intact uterus to prevent endometrial cancer; however, its inclusion in the HRT formulation has been found to increase the incidence of breast cancer significantly compared with that in post-menopausal women undergoing HRT containing estrogen alone (Ross et al. 2000; Chlebowski et al. 2003; Writing Group for the Women’s Health Initiative Investigators 2002). The increased risk of breast cancer is greatly reduced after progestin use ceases, correlating with a 7 % decline in breast cancer incidence after the results of the WHI trial were announced (Siegel et al. 2015; Writing Group for the Women’s Health Initiative Investigators 2002; Tsai et al. 2011). Although the use of HRT has become increasingly controversial, in the United States an estimated 1.6 million women take combined estrogen/progestin HRT to alleviate the symptoms of menopause (Tsai et al. 2011).

Studies from our laboratory and others have shown that progestins promote the development of hormone-responsive breast cancers by increasing the production of vascular endothelial growth factor (VEGF). This in turn increases neovascularization, cell proliferation, and metastasis (Folkman 1995; Hanahan and Folkman 1996; Liang and Hyder 2005; Liang et al. 2010). The synthetic anti-progestin RU-486, which acts by blocking the progesterone receptor (PR), inhibits progestin-dependent tumor growth, indicating that the process is dependent on PR. We also have shown that progestins stimulate breast cancer metastasis to lymph nodes in an animal model, a phenomenon that has recently been confirmed in human subjects (Liang et al. 2010; Chlebowski et al. 2009). Due to the rapid onset of HRT-driven tumors and the similarities between metastatic cancer cells and stem cell-like cells, it has been suggested that latent cells that are not normally exposed to progestins are revitalized by exogenous progestin (Hyder et al. 1998; Horwitz and Sartorius 2008; Brisken 2013). This stimulation causes increased proliferation and leads ultimately to a more aggressive phenotype (Liang and Hyder 2005; Liang et al. 2010). In support of this concept, progestins have been shown to enrich the stem cell-like cancer cell population in vitro by dedifferentiating progenitor cells back to a stem cell-like origin (Horwitz and Sartorius 2008; Brisken 2013). Thus, progestins appear to promote breast cancer not only by increasing production of the potent mitogenic factor VEGF and stimulating tumor and endothelial cell proliferation (Liang and Hyder 2005), but also by enriching the stem cell-like population, thereby enabling tumors to grow and metastasize (Liang et al. 2010; Horwitz et al. 2013).

Most synthetic ligands with anti-progestin properties are toxic and cross-react with other steroid receptors, preventing their long-term use (Horwitz 1992). In contrast, the majority of naturally occurring compounds are non-toxic. We therefore undertook studies to identify natural compounds with anti-progestin-like activities that might be used to counter the pro-tumor effects of progestins. Luteolin (LU), a flavonoid that is found in more than 300 plant species (many of which are readily available in the human diet), has recently been shown to inhibit a variety of cancers, both in vitro and in vivo, with little to minimal toxicity (Seelinger et al. 2008). Previously we have shown that LU prevents and delays medroxyprogesterone acetate (MPA)-dependent tumor development in the 7,12-dimethylbenz(A)anthracene-induced tumor model, and we proposed that LU possesses long-lasting anti-cancer effects (manuscript under review). In the present study, we conducted studies in a xenograft model to determine whether LU might also be used to treat progestin-dependent breast cancer (Liang et al. 2007). Herein we demonstrate that LU effectively blocks the growth of progestin-dependent human xenograft tumors, inhibits angiogenesis, and restricts the conversion of breast cancer cells into stem cell-like cells.

## Methods

### Reagents

Luteolin (2-(3,4-dihydroxyphenyl)-5,7-dihydroxy-4H-1-benzopyran-4-one) (LU) was purchased from Tocris (Minneapolis, MN, USA) and dissolved in sterile filtered dimethyl sulfoxide (DMSO; Sigma-Aldrich; St. Louis, MO, USA). Medroxyprogesterone acetate (MPA), progesterone, norethindrone, norgestrel, and RU-486 were purchased from Sigma-Aldrich. Pierce bicinchoninic acid protein reagents were obtained from Fisher Scientific (Pittsburgh, PA, USA). 17-β estradiol (E2; 1.7 mg), MPA (10 mg), and placebo 60-day release pellets were obtained from Innovative Research of America (Sarasota, FL, USA).

### Cell lines and culture

Hormone-responsive BT-474 and T47-D human breast cancer cell lines were obtained from the American Type Culture Collection (Manassas, VA, USA) and maintained at 37 °C in phenol red-free DMEM/F12 medium (Invitrogen, Waltham, MA, USA) supplemented with 10 % fetal bovine serum (FBS; Sigma-Aldrich) in a humidified atmosphere of 5 % CO_2_. For all in vitro experiments, cells were maintained in DMEM/F12 supplemented with 5 % dextran-coated charcoal (DCC)-stripped FBS for 24 h prior to treatment. Subsequently, cells were washed and further incubated in fresh 5 % DCC-stripped FBS-DMEM/F12.

### Cell viability assay

Viable cells were quantitated using sulforhodamine B (SRB) assays (Skehan et al. 1990). In brief, breast cancer cells in 100 µl DMEM/F12/10 % FBS medium were seeded into each well of a 96-well plate and incubated at 37 °C overnight in 5 % CO_2_. Cells were treated (in six replicates) with either LU or DMSO (controls) in DMEM/5 % FBS for periods up to 48 h, then subjected to SRB assays.

### Apoptosis assay

Apoptosis was evaluated by staining with Annexin V-fluorescein isothiocyanate (FITC) and propidium iodide (PI) as described previously (Liang et al. 2006). T47-D cells were grown to 50–60 % confluence in DMEM/F12/10 % FBS, at which point the media was switched to 5 % DCC-stripped FBS-DMEM/F12. After 24 h, cells were treated with LU ± MPA for an additional 16 h. Treated cells were harvested using 0.05 % trypsin–EDTA, stained, and subjected for fluorescence-activated cell sorting (FACS) analysis per the manufacturer’s protocol (BioVision Inc, Milpitas, CA, USA).

### VEGF enzyme-linked immunosorbent assay (ELISA)

The Quantikine human VEGF ELISA kit was purchased from R&D Systems, Inc. (Minneapolis, MN, USA). Supernatant from treated cells was collected and VEGF concentrations measured according to the manufacturer’s protocol. Experiments were performed in triplicate, and each sample was analyzed in duplicate on a microplate reader. Inter- and intra-assay coefficients of variance given by the manufacturer for cell culture supernatant assays are 5–8.5 and 3.5–6.5 %, respectively.

### Bicinchoninic acid protein assay

Cells were harvested and pellets resuspended in 300 µl lysis buffer (50 mM Tris/HCl, pH 8, 150 mM NaCl, and 1 % Nonidet P-40). Protein concentration was determined by measuring absorbance at 562 nm on a microplate reader, using bovine serum albumin (Thermo Fisher Scientific; Waltham, MA, USA) as standard. Experiments were performed in triplicate, and samples were analyzed in duplicate.

### Reverse transcription-polymerase chain reaction (RT-PCR)

RNA from progestin-treated cells was purified and RT-PCR conducted as described previously (Mafuvadze et al. 2010). The primers used were:

VEGF

$${\text{F}} \quad 5^{\prime} {\text{-}} {\text{CTGCTGTCTTGGGTGCATTGG}}$$

$${\text{R}} \quad 5^{\prime} {\text{-}} {\text{CACCGCCTCGGCTTGTCACAT}}$$

Glyceraldehyde phosphate dehydrogenase (GAPDH)

$${\text{F}} \quad 5^{\prime} {\text{-}} {\text{ATGAGA AGTATGACAGCC}}$$

$${\text{R}} \quad 5^{\prime} {\text{-}} {\text{TGAGTCCTTCCACGATACC}}$$

### FACS analyses

For all FACS analysis, treated breast cancer cells were harvested using Accutase (BD Biosciences; San Jose, CA, USA) in place of trypsin–EDTA. Cells (1 × 10^6^) were then suspended in 100 µl staining buffer and placed in microcentrifuge tubes.

Phycoerythrin (PE)-conjugated mouse anti-human CD24 (20 µl) and allophycocyanin (APC)-conjugated mouse anti-human CD44 (20 µl) antibodies (both from BD Biosciences) were added to each sample, along with the necessary FACS dye controls, and the samples incubated on ice for 45 min. Cells were washed twice in staining buffer, resuspended in 500 µl staining buffer and 1 μl of 250 μg/ml PI, and subjected to FACS analysis.

Aldehyde dehydrogenase (ALDH) activity was assessed using the ALDEFLUOR kit (STEMCELL Technologies Inc.; Vancouver, BC, Canada), according to the manufacturer’s protocol. All samples were processed within 15 min of the final wash. Cells were visualized using a Beckman Coulter CyAn ADP FACS machine running Summit 5.2 software and results analyzed as previously described (Ginestier et al. 2007).

### Mammosphere-formation assay

T47-D cells were grown in 10 % FBS DMEM/F12 medium, then cultured in 5 % DCC-stripped FBS-DMEM/F12 medium for 24 h. Cells were then treated for 48 h with indicated agent(s) of interest. Cells from each group were subsequently seeded into six-well plates (5000 cells/well) in Complete MammoCult medium (STEMCELL Technologies Inc.) and treatment continued for six more days. Culture medium (1 ml) was refreshed on days 2, 4, and 6 to ensure drug availability. Light microscopy (10×) pictures of mammospheres formed were captured after 7 days using an EVOS light microscope. Mammospheres ≥60 µm in diameter (determined by size exclusion) were counted.

### Human breast cancer cell xenograft studies

Xenograft experiments were performed as described previously (Liang et al. 2007). All facilities were approved by the American Association for Accreditation of Laboratory Animal Care in accordance with current federal regulations and standards. In brief, an E2 60-day release pellet (1.7 mg) was implanted in each nude mouse. Two days later, T47-D cells were suspended in DMEM/F12 medium and injected subcutaneously (1 × 10^7^ cells per 150 µl) into each flank of nude mouse (n = 2–4 animals/group). Mice were then implanted with a 60-day release MPA (10 mg) or placebo pellet 10 days after breast cancer cell injection. When tumors reached about 60 mm^3^, intraperitoneal treatment with LU (20 mg/kg/day) or vehicle commenced. LU was administered daily for 2 days (loading dose), then every other day until day 79.

### Immunohistochemical analyses

Xenograft tumor-bearing mice were sacrificed at day 79 and tumors collected and processed for immunohistochemical analysis as previously described (Liang et al. 2007). Tumors were collected from both flanks of each mouse and at least three tumors per group were collected for analysis. One section from each individual tumor was subjected to immunohistochemical staining using anti-VEGF (1:100 dilution; Santa Cruz Biotechnology; Dallas, TX, USA), anti-PR, or anti-CD31 polyclonal antibodies, both at 1:50 dilution (DAKO; Carpinteria, CA, USA).

Four random fields were captured from every stained section to minimize errors due to differences in cellularity. Regions of staining within tumors were recorded. Fovea Pro 3.0 software (Reindeer Graphics; Ashville, NC, USA) was used to quantitate the percent area of VEGF staining, while the percent of PR-positively stained cells was calculated using the color threshold in Image J (NIH). This facilitated precise discrimination between positive/negative cells and background. For quantitating blood vessels, five CD31-labeled 10x sections were taken from each tumor to minimize intra-tumoral variation (Liang et al. 2007). The total number of vessels was counted in each section and then averaged per corresponding tumor.

Immunohistochemical staining data were reported as mean ± standard error of the mean (SEM) per treatment group, with each group having an n ≥ 3 tumors analyzed.

### Statistical analysis

Statistical significance was tested using one-way analysis of variance (ANOVA) followed by a Newman–Keuls multiple comparison test to determine the difference in mean between groups. A two-way repeated measures ANOVA was used for animal weights. If normality failed, the data were tested using a nonparametric one-way ANOVA on ranks (Kruskal–Wallis), followed by Newman–Keuls comparison test. Data were reported as mean ± SEM. For all comparisons, *P* ≤ 0.05 was regarded as statistically significant. Analyses were performed using SigmaPlot 12.5 software.

## Results

### Luteolin reduces viability of human breast cancer cells

**Fig. 1:**
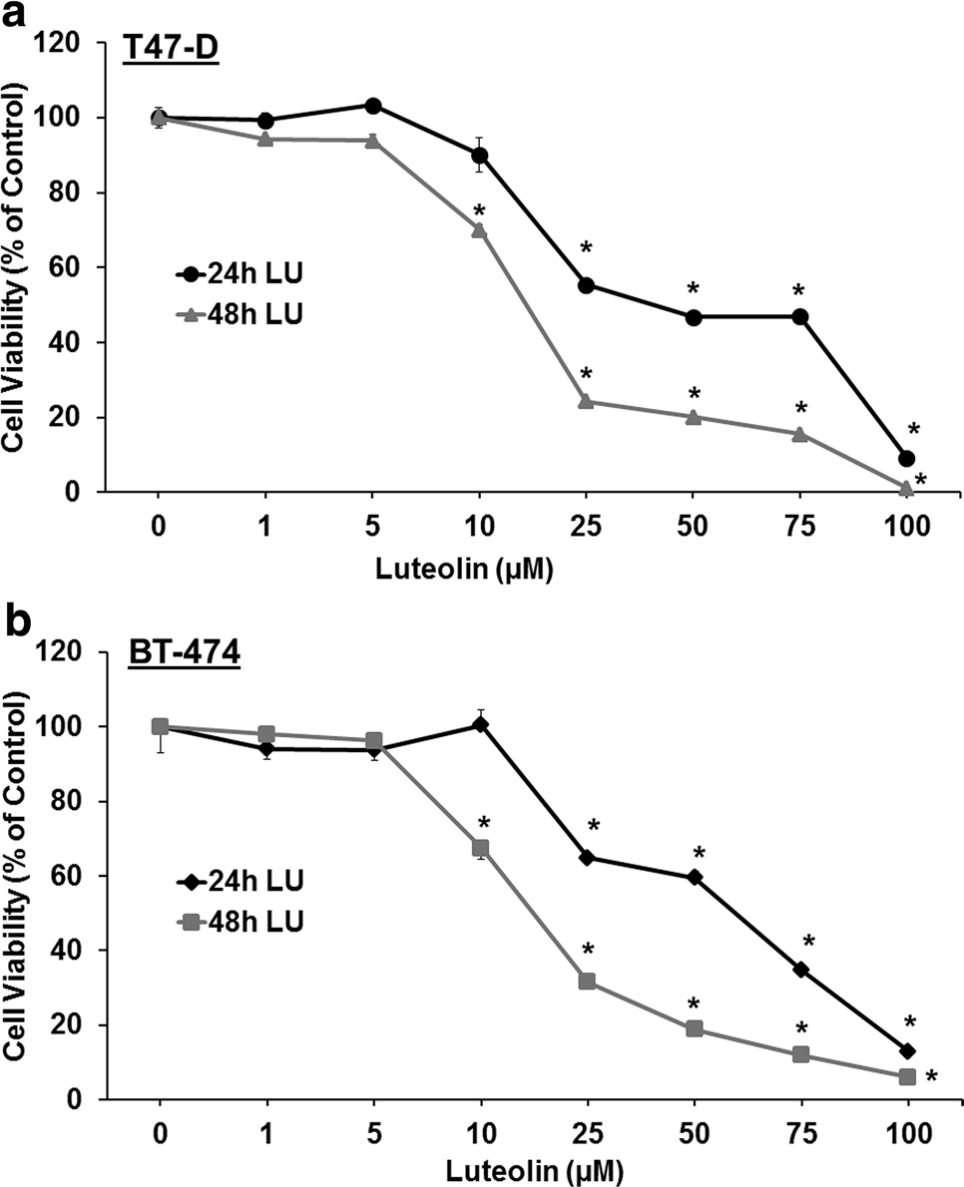
Luteolin reduces human breast cancer cell viability. T47-D (**a**) and BT-474 (**b**) breast cancer cells were seeded overnight in a 96-well plate (0.5 × 10^4^ cells/well and 1 × 10^4^ cells/well, respectively). Cells were then washed and treated with the indicated concentration of luteolin (LU) for either 24 or 48 h. Cell viability was determined using SRB assays. Results represent mean ± SEM (n = 3, in six replicates). *Significantly different from control (DMSO only) (*P* < 0.05, ANOVA)

When two estrogen receptor- and PR-positive human breast cancer cell lines (BT-474 and T47-D) were exposed for 24 or 48 h to varying concentrations of LU (0–100 μM) used in previous studies (Seelinger et al. 2008), LU markedly reduced cell viability in both a time- and dose-dependent manner (Fig. 1a, b). Following 24-h exposure, LU exhibited an IC_50_ value of approximately 50 μM against these cells, with minimal or no effect observed at 10 μM during the same treatment period. Thus, unless otherwise stated, subsequent experiments were conducted with 10 μM LU for 16–18 h in order to determine the biological effects of LU without inducing loss of cell viability and cell death.

### Luteolin inhibits progestin-induced VEGF secretion from breast cancer cells

**Fig. 2:**
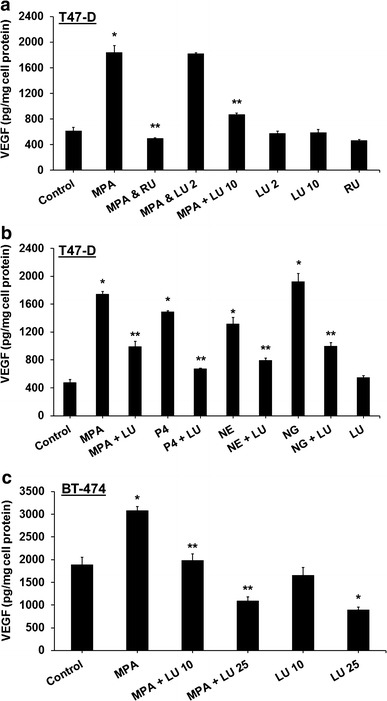
Luteolin inhibits progestin-induced secretion of VEGF from human breast cancer cells. **a** T47-D cells were treated at 37 °C for 18 h with 10 nM MPA, 10 nM MPA + 1 µM RU-486 (RU), 10 nM MPA + 2 or 10 µM luteolin (LU), 2 µM or 10 µM LU, or 1 µM RU-486. **b** T47-D cells were treated at 37 °C for 18 h with 10 nM MPA, progesterone (P4), norethindrone (NE), or norgestrel (NG) ± 10 µM LU, or 10 µM LU alone. **c** BT-474 cells were treated at 37 °C for 18 h with 10 nM MPA ± 10 or 25 µM LU, or 10 µM or 25 µM LU alone. Levels of VEGF in culture medium were measured by ELISA. In all experiments, LU or RU was administered 30 min prior to MPA. VEGF ELISA values were normalized to cellular protein content, measured using bicinchoninic acid protein assays. Results represent mean ± SEM (n = 3, in duplicate). *Significantly different from control (DMSO only) (*P* < 0.05, ANOVA). **Significantly different from progestin alone (*P* < 0.05, ANOVA)

We have previously established that both natural and synthetic progestins induce synthesis and secretion of the potent angiogenic factor VEGF from both T47-D and BT-474 cells (Liang and Hyder 2005; Hyder et al. 1998). To examine LU’s ability to block progestin-induced secretion of VEGF from breast cancer cells, T47-D cells were treated for 18 h with MPA, both with and without LU or RU-486, then release of VEGF into the culture medium measured. Treatment with MPA significantly increased VEGF secretion. Levels of MPA-dependent VEGF secretion were significantly reduced by 10 µM LU; however, 2 µM LU had no effect (Fig. 2a). Similarly, 10 µM LU lowered VEGF secretion in response to both progesterone and two commonly used synthetic progestins, norethindrone and norgestrel (Fig. 2b). Importantly, LU alone did not induce VEGF, therefore behaving in a similar fashion to RU-486 (Fig. 2a).

When we examined whether LU exerted similar effects in other breast cancer cells, we found that LU (10 µM) also significantly reduced levels of MPA-induced VEGF secretion in BT-474 cells. At higher concentrations (25 µM), LU blocked even basal levels of VEGF secretion (Fig. 2c).

### Luteolin suppresses progestin-induced VEGF mRNA expression in breast cancer cells

**Fig. 3:**
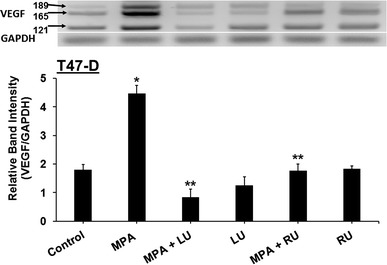
Luteolin inhibits MPA-induced VEGF mRNA expression in T47-D breast cancer cells. T47-D cells were treated at 37 °C for 6 h with 10 nM MPA ± 25 µM luteolin (LU) or 1 µM RU-486 (RU), or 25 µM LU or 1 µM RU-486 alone, after which RNA was isolated and RT-PCR for VEGF isoforms performed. *Upper panel* Representative figure of PCR-amplified VEGF products, showing VEGF 189, 165, and 121 bp bands and the GAPDH band used for normalization. *Lower panel* Results represent mean band intensities (VEGF/GAPDH) ± SEM (n = 3). *Significantly different from control (DMSO only) (*P* < 0.001, ANOVA). **Significantly different from MPA (*P* < 0.001, ANOVA)

When T47-D cells were used to determine whether LU suppressed progestin-induced VEGF at the mRNA level, MPA induced VEGF mRNA isoforms, while LU and RU-486 both suppressed MPA-induced VEGF mRNA expression. Neither LU nor RU-486 alone induced VEGF mRNA expression (Fig. 3).

### Luteolin induces apoptosis in breast cancer cells

**Fig. 4:**
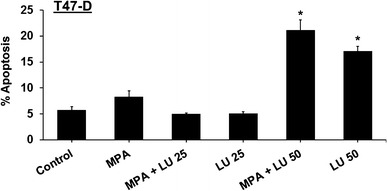
Luteolin induces apoptosis in breast cancer cells. T47-D cells were treated with 10 nM MPA ± 25 or 50 µM luteolin (LU), or 25 or 50 µM LU alone at 37 °C for 16 h. Cells were stained with Annexin V-FITC and PI and analyzed by FACS. Results represent mean ± SEM (n = 3). *Significantly different from control (DMSO only) (*P* < 0.001, ANOVA)

When T47-D cells were used to determine whether LU induced breast cancer cell apoptosis, 50 µM LU induced apoptosis whether or not MPA was present (Fig. 4), indicating that MPA was unable to protect breast cancer cells from LU-induced cell death.

### Luteolin inhibits MPA-induced breast cancer cell xenograft tumor growth in vivo

**Fig. 5:**
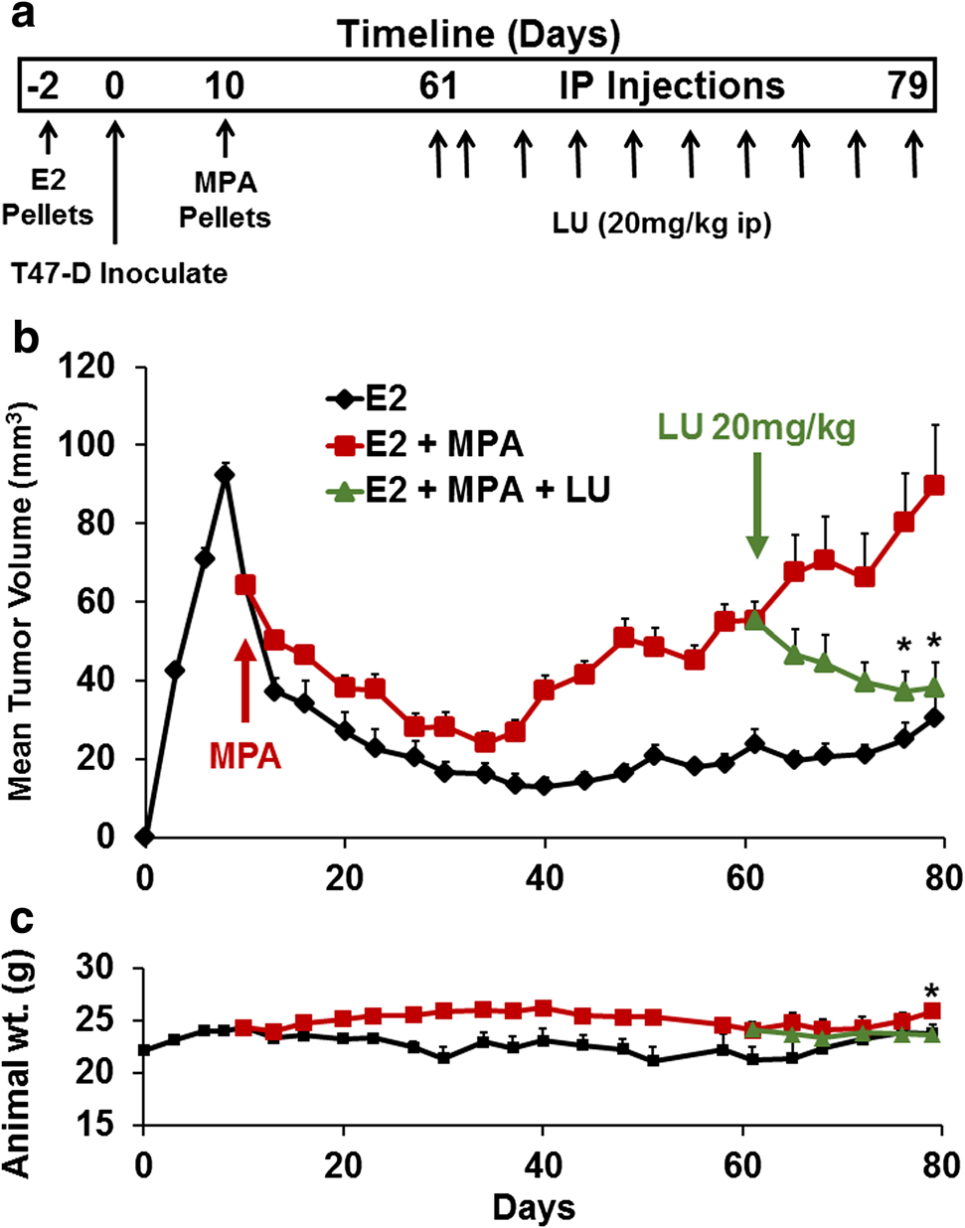
Luteolin suppresses in vivo growth of MPA-accelerated human T47-D breast cancer cells in a xenograft model. **a** Protocol for xenograft tumor growth and treatment. An estradiol (E2) pellet was implanted in nude mice and, 2 days later, T47-D cells (1 × 10^7^) were injected subcutaneously into each flank of nude mouse. MPA (10 mg) or placebo pellets were implanted on day 10. When tumors reached approximately 60 mm^3^, treatment with luteolin (LU) (20 mg/kg) or vehicle began (*arrow* day 61). LU was injected intraperitoneal (ip) daily for 2 days (loading dose), followed by injections every other day until day 79. **b** Luteolin suppresses xenograft tumor growth in vivo. Mice were palpated and tumors measured every other day, and tumor volumes calculated as described (Liang et al. 2007). Results represent mean tumor volumes ± SEM [E2 group (E2 pellet + vehicle), n = 3 tumors; E2 + MPA group (MPA pellet + vehicle), n = 7 tumors; E2 + MPA + LU group (MPA pellet + LU), n = 8 tumors]. *Significantly different from MPA (*P* < 0.05, ANOVA). **c** Luteolin does not affect animal weights throughout the experiment. Animals were weighed twice weekly. Results represent mean weights ± SEM. *Significantly different from E2 (*P* < 0.05, ANOVA)

We next studied LU’s therapeutic effect in a progestin-dependent T47-D xenograft tumor model previously developed in our laboratory (Liang et al. 2007). The experimental protocol is shown in Fig. 5a. LU blocked progestin-induced T47-D tumor growth; tumor volumes in LU-treated animals decreased to those of control animals by day 76 (Fig. 5b). No LU-related toxicity was observed in any of the experimental animals, as determined by animal weight (Fig. 5c). In addition, animal behavior (i.e., eating, grooming, and mobility) was no different in LU-treated mice, further suggesting that LU had little to no toxicity.

### Luteolin reduces expression of angiogenesis markers in breast cancer cell xenograft tumors

**Fig. 6:**
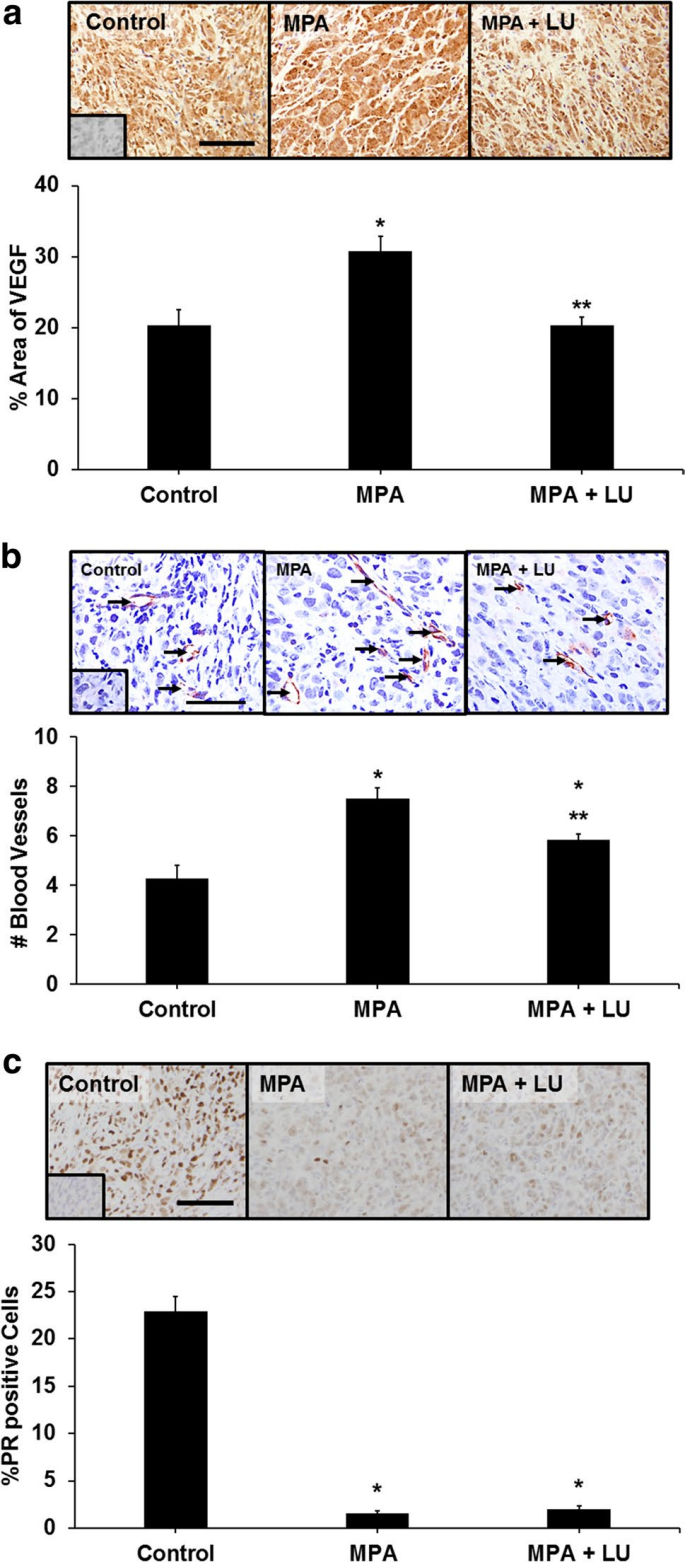
Luteolin reduces expression of angiogenesis markers and PR in breast cancer cell xenograft tumors. **a** Luteolin suppresses MPA-driven VEGF expression in T47-D xenografts. Xenograft tumor-bearing mice were sacrificed at day 79 and tumors collected, processed, and subjected to immunohistochemistry. *Upper panel* Images represent VEGF (*brown*) staining from one tumor per group. *Scale bar* 100 µM. *Lower panel* Results represent quantification of VEGF staining (mean ± SEM percent area of staining) [control (placebo pellet + vehicle), n = 3 tumors; MPA (MPA pellet + vehicle), n = 7 tumors; MPA + luteolin (LU) (MPA pellet + LU), n = 8 tumors]. *Significantly different from control (*P* = 0.007, ANOVA). **Significantly different from MPA (*P* < 0.001; ANOVA). *Inset* represents no antibody control. **b** Luteolin suppresses MPA-driven increases in blood-vessel density in T47-D xenografts. *Upper panel* Images represent CD31 endothelial staining (*reddish-brown*) of blood vessels from one tumor per group from sections of a ×20 field at captured resolution. *Scale bar* 50 µm. *Lower panel* Results represent quantitation of number of blood vessels stained. Five captures at ×20 were taken per tumor in each group [control (E2 pellet + vehicle), n = 3 tumors; MPA (MPA pellet + vehicle), n = 7 tumors; MPA + LU (MPA pellet + LU), n = 8 tumors]. The number of blood vessels was counted in each tumor capture, averaged for each individual tumor, and the data represent mean number of blood vessels/tumor capture ± SEM. *Significantly different from control (*P* < 0.001, ANOVA). **Significantly different from control and MPA alone (*P* = 0.003, ANOVA followed by a Newman–Keuls multiple comparison test). *Inset* represents no antibody control. *Arrows* point to blood vessels represented by CD-31 staining. **c** Luteolin does not restore MPA-driven loss of PR expression in T47-D xenografts. *Upper panel* Images represent PR staining from one tumor per group [control (placebo pellet + vehicle), n = 3 tumors; MPA (MPA pellet + vehicle), n = 7 tumors; MPA + LU (MPA pellet + LU), n = 8 tumors]. *Scale bar* 100 µm. *Lower panel* Results represent quantification of the percent of PR-positively stained cells, means + SEM. *Significantly different from control [*P* < 0.05, ANOVA on ranks (Kruskal–Wallis), followed by the Newman–Keuls nonparametric multiple comparisons test]. *Inset* represents no antibody control

We have previously shown that progestins increase tumor burden by inducing the angiogenic factor VEGF, suggesting that the tumor growth observed in those studies was most likely due to increased angiogenesis (Liang et al. 2007, 2010). In the present study, when our xenograft model was used to examine the ability of LU to block MPA-driven VEGF induction, tumor VEGF expression was significantly reduced in MPA + LU-treated animals compared with animals given MPA alone (Fig. 6a). Similarly, animals treated with MPA + LU demonstrated significantly reduced tumor blood-vessel density compared with animals administered MPA alone (Fig. 6b).

### Luteolin does not prevent MPA-induced loss of PR in breast cancer cell xenograft tumors

Xenograft tumor tissues demonstrated an almost complete loss of PR in animals given MPA alone, concurring with previous reports that this represents an active PR function (Knutson and Lange 2014). LU treatment did not prevent the MPA-induced loss of PR in xenograft tumors (Fig. 6c), suggesting that it does not block PR activation, but rather acts at a point beyond the PR activation step or exerts other post-transcriptional effects on VEGF mRNA or protein. The inability of LU to rescue PR expression was verified by Western-blot analysis of tumor cells in vitro, in which MPA was again shown to lower PR protein expression, whether LU was present or not (data not shown).

### Luteolin inhibits MPA-induced stem cell-like properties of breast cancer cells

**Fig. 7:**
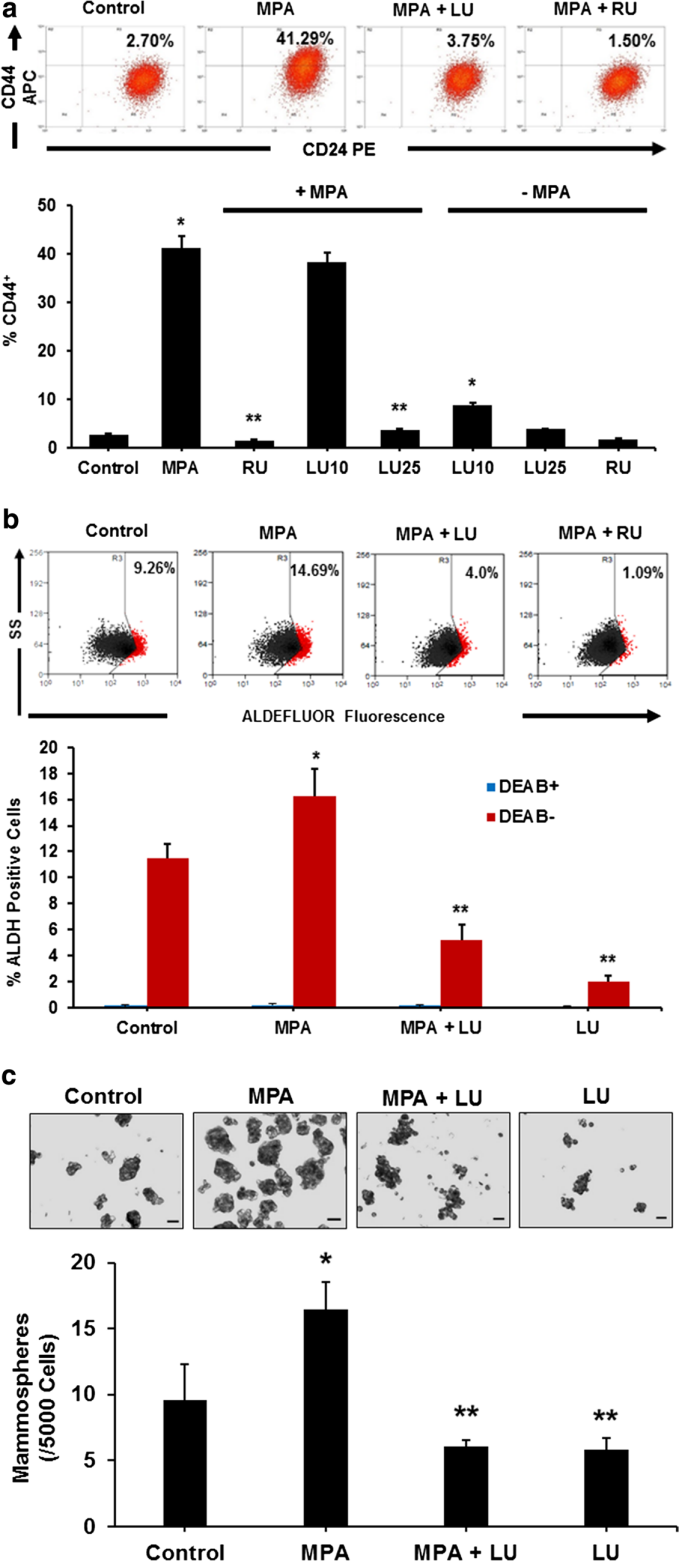
Luteolin suppresses MPA-induced stem cell-like properties of T47-D breast cancer cells. **a**, **b** Luteolin suppresses MPA-induced breast cancer cell CD44 expression. T47-D cells were incubated at 37 °C for 24 h with 10 nM MPA, 10 or 25 µM luteolin (LU), 1 µM RU-486 (RU), or 10 nM MPA + 10 µM LU, 25 µM, or 1 µM RU-486. Following treatment, cells were harvested and labeled with CD44-APC and CD24-PE antibodies and analyzed by FACS. **a** *Upper panel* Displays flow cytometry data for control (DMSO only), 10 nM MPA, 10 nM MPA + 25 µM LU, and 10 nM MPA + 1 µM RU-486. **b** *Lower panel* Quantitative data from three different FACS analysis experiments. Results represent mean ± SEM (n = 3). *Significantly different from control. **Significantly different from MPA (*P* < 0.05, ANOVA). **b** Luteolin inhibits MPA-induced ALDH^bright^ induction. T47-D cells were treated with 10 nM MPA ± 25 µM LU or 25 µM LU alone at 37 °C for 24 h. Following treatment, cells were harvested and ALDH activity measured by FACS analysis using the ALDEFLUOR assay. *Upper panel* Representative flow cytometry data; ALDH^bright^ cells are shown in *red*. The ALDH^bright^ gate was set using negative controls DEAB+. Viable cells were gated using PI; once the gates were set, they were applied throughout the analyses. *Lower panel* Quantitative data from four different ALDEFLUOR assay experiments. Results represent mean ± SEM (n = 4). *Significantly different from control (DMSO only). **Significantly different from control and MPA (*P* < 0.05, ANOVA followed by a Newman–Keuls multiple comparison test). **c** Luteolin suppresses MPA-induced breast cancer cell mammosphere formation. T47-D cells were treated at 37 °C for 48 h with 10 nM MPA, 10 nM MPA + 25 µM LU, or 25 µM LU alone. Following treatment, cells from each group were seeded into six-well plates (5000 cells/well) and treated for seven more days in Complete MammoCult medium. Cells were re-treated with the agent(s) of interest in 1 ml culture medium on days 2, 4, and 6. *Upper panel* Representative light microscopic images of T47-D mammospheres formed after 7 days. *Scale bar* 60 μm. *Lower panel* Results represent mean number of mammospheres ± SEM (n = 3). Number of mammospheres ≥60 μm was quantitated from 6 to 9 images per well, three wells per group. *Significantly different from control (DMSO only) (*P* < 0.05); **Significantly different from MPA (*P* < 0.05, ANOVA on ranks followed by Dunn’s method of multiple comparisons)

We have previously shown that MPA stimulates in vivo tumor cell growth, a phenomenon that is likely linked to its ability to enrich the stem cell-like properties in a small subportion of tumor cells (Hyder et al. 1998; Horwitz and Sartorius 2008). In this study, we examined LU effects on MPA-induced acquisition of stem cell-like properties of breast cancer cells using three indicators of the stem-cell phenotype. First, FACS analysis of CD44, a well-recognized marker of breast cancer stem cells, demonstrated that MPA induced a large and highly reproducible CD44^+^ shift in T47-D cells, suggesting that MPA induces an increase in stem cell- or progenitor-like cells, as previously shown (Horwitz and Sartorius 2008; Al-Hajj et al. 2003; Axlund and Sartorius 2012). The MPA-induced increase in the CD44^+^ population was significantly reduced by exposure to either 25 µM LU or 1 µM RU-486 (Fig. 7a). The LU effects on MPA induction of CD44 were dose-dependent, given that 25 µM LU + MPA but not 10 µM LU + MPA significantly decreased the MPA-induced increase in the CD44^+^ population (Fig. 7a).

Next, MPA induced a significant increase in ALDH^bright^ activity, which is another established stem-cell marker in a variety of cancers, including breast cancer (Ginestier et al. 2007), whereas LU + MPA treatment significantly reduced ALDH^bright^ activity compared with that observed for MPA treatment alone. LU treatment alone also significantly reduced basal ALDH^bright^ activity (Fig. 7b), suggesting that LU has an inherent ability to reduce the stem cell-like properties of breast cancer cells.

Lastly, mammosphere-formation assays, in which only stem cells with self-renewal capability are able to seed mammospheres in an anchorage-independent three-dimensional environment (Liu et al. 2005), demonstrated that MPA treatment alone caused a significant increase in the number of mammospheres formed by T47-D cells, an effect that was blunted when cells were treated with MPA + LU. Further, LU treatment alone did not increase the number of mammospheres (Fig. 7c).

## Discussion

A number of recent clinical trials and studies have shown that use of estrogen/progestin combination HRT regimens to alleviate the symptoms of menopause leads to a significantly increased breast cancer risk (Writing Group for the Women’s Health Initiative Investigators 2002; MWS Collaborators 2003). Investigators have attempted to understand the role of progestins in this process. Earlier studies from our laboratory have shown that induction of the potently angiogenic VEGF in both T47-D and BT-474 cells is one possible mechanism that might explain increased incidence of breast cancer arising in response to combination HRT (Liang and Hyder 2005; Hyder et al. 1998). Other mechanisms, such as progestin-dependent increases in tumor cell proliferation and development of stem cell-like properties by tumor cells, have also been suggested (Liang and Hyder 2005; Horwitz and Sartorius 2008). Because adjuncts to stem the pro-tumor effects of combination HRT are needed, in the present study we examined the therapeutic ability of the naturally occurring non-toxic flavonoid LU to suppress the growth of and expression of angiogenesis markers in progestin-dependent human breast cancer cell xenograft tumors in vivo, as well as it ability to suppress VEGF induction and the stem cell-like phenotype of breast cancer cells in vitro.

In this study, LU blocked progestin-induced VEGF secretion in both T47-D and BT-474 cells in a similar fashion as apigenin, another naturally occurring flavonoid tested in our laboratory (Mafuvadze et al. 2012). It appears, however, that LU might be a superior pharmacologically active compound to apigenin due to low potential of metabolism into potentially toxic compounds (Seelinger et al. 2008). LU blocked VEGF secretion stimulated by both natural and synthetic progestins, including MPA, norgestrel, and norethindrone, all of which are common components of HRT in the United States and Europe. Furthermore, LU suppressed progestin-induced VEGF mRNA expression. Interestingly, LU had no effect on PR at either the mRNA or protein level (data not shown), suggesting that it may interfere with the interaction of PR at PRE on the VEGF promoter (Hyder et al. 2000), or act at a downstream step by modifying co-activators needed for PR-dependent gene transcription (Wu et al. 2004), though these possibilities remain to be tested. LU’s ability to block progestin-induced VEGF production in human breast cancer cells could also be due to suppression of the phosphoinositide-3′-kinase pathway (Bagli et al. 2004), or inhibition of the SP-1 transcription factor, both of which are known to control progestin-induced VEGF induction in human breast cancer cells (Wu et al. 2004). These possibilities also remain to be tested.

In T47-D xenografts, LU treatment reduced tumor growth and, in fact, caused tumor regression, likely due to inhibition of MPA-induced VEGF secretion from tumor cells. Because VEGF has also been shown to protect cells from undergoing apoptosis (Liang et al. 2006), loss of VEGF may increase tumor cell apoptosis as well as inhibit angiogenesis. We observed a decrease in the number of tumor blood vessels following LU treatment; however, this reduction in tumor volume was not associated with increased apoptosis or decreased cell proliferation (data not shown), suggesting that neither mechanism is involved in the LU effects on tumor growth.

Luteolin suppressed progestin-mediated increases in stem cell-like properties of breast cancer cells in established assays examining CD44 expression, mammosphere formation, and ALDH^bright^ activity. Cells that have increased CD44 have been shown to be more aggressive in terms of growth and motility (Al-Hajj et al. 2003), and could be responsible for increased tumor growth seen in our established model (Liang et al. 2007). These data suggest that LU reduces the number of cancer stem cell-like cells and/or progenitor cells in progestin-responsive breast cancer and may thereby be able to reverse tumor growth (Liang and Hyder 2005; Horwitz and Sartorius 2008; Cittelly et al. 2013).

## Conclusions

In summary, our studies provide evidence that LU has the potential to disrupt angiogenesis and thereby prevent the growth development of progestin-driven tumors. They also provide evidence that LU reduced the MPA-driven cancer stem cell-like and/or progenitor cell subpopulation, strongly suggesting that it exerts its anti-tumor effects in a variety of ways. LU demonstrates significant potential as a new and novel agent that might be used to combat particularly aggressive and hard-to-treat types of breast cancer. It is therefore essential that we further investigate the mechanisms by which LU moderates progestin effects in order to fully exploit its therapeutic potential.

## Abbreviations

HRT: hormone replacement therapy
VEGF: vascular endothelial growth factor
PR: progesterone receptor
LU: luteolin
DMSO: dimethyl sulfoxide
MPA: medroxyprogesterone acetate
E2: Estradiol
FBS: fetal bovine serum
DCC: dextran-coated charcoal
SRB: sulforhodamine B
FITC: fluorescein isothiocyanate
PI: propidium iodide
FACS: fluorescence-activated cell sorting
ELISA: enzyme-linked immunosorbent assay
RT-PCR: reverse transcription-polymerase chain reaction
GAPDH: glyceraldehyde phosphate dehydrogenase
ALDH: aldehyde dehydrogenase
PE: phycoerythrin
APC: allophycocyanin
SEM: standard error of the mean
ANOVA: analysis of variance
